# Immune-Related Gene Expression Analysis Revealed Three lncRNAs as Prognostic Factors for Colon Cancer

**DOI:** 10.3389/fgene.2021.690053

**Published:** 2021-07-09

**Authors:** Xiao-Liang Xing, Ti Zhang, Zhi-Yong Yao, Chaoqun Xing, Chunxiao Wang, Yuan-Wu Liu, Minjiang Huang

**Affiliations:** ^1^School of Public Health and Laboratory Medicine, Hunan University of Medicine, Huaihua, China; ^2^Beijing Advanced Innovation Center for Food Nutrition and Human Health, China Agricultural University, Beijing, China

**Keywords:** COAD, immune, lncRNAs, overall survival, risk model

## Abstract

Colorectal cancer (CRC) is one of the most common cancers. Almost 80% of CRC cases are colon adenocarcinomas (COADs). Several studies have indicated the role of immunotherapy in the treatment of various cancers. Our study aimed to identify immune-related long non-coding RNAs (lncRNAs) and to use them to construct a risk assessment model for evaluating COAD prognosis. Using differential expression, correlation, and Cox regression analyses, we identified three immune-related differentially expressed lncRNAs (IR-DELs) and used them to construct a risk assessment model. The area under the curve (AUC) for each receiver operating characteristic (ROC) curve at 3-, 5-, and 10-years were greater than 0.6. In addition, the risk assessment model was correlated with several immune cells and factors. The three IR-DELs (AC124067.4, LINC02604, and MIR4435-2HG) identified in this study can be used to predict outcomes for patients with COAD and might help in identifying those who can benefit from anti-tumor immunotherapy.

## Introduction

Colorectal cancer (CRC) is the third most common cancer worldwide, with nearly 1.8 million new cases and 861,000 deaths reported in 2018 (Bray et al., 2018). Depending on its anatomical location, CRC can be classified either as colon adenocarcinoma (COAD) or rectal adenocarcinoma (READ). COAD accounts for approximately 80% of CRC cases, and almost 50% patients with CRC will develop distant metastases (Arnold et al., 2017). The standard methods for CRC treatment are surgery, chemotherapy, and radiotherapy. These treatments can be combined, depending on the location and progression of the cancer (Johdi and Sukor, 2020). For example, approximately 66–61% of stage II and stage III patients with colon and rectal cancer undergo further treatments with adjuvant chemotherapy and/or radiotherapy, respectively (Miller et al., 2019). Approximately 50% of patients relapse even after additional treatment with neoadjuvant therapy (Ogura et al., 2019). Even with combination therapy, the overall survival (OS) rate of patients with advanced COAD remains low.

Cancer immunotherapy targets specific cancer antigens on the malignant cells, alerting the immune system to eradicate cancer through concerted immune responses (Johdi and Sukor, 2020). Immune cells and factors have antitumor effects, including antitumor initiation and progression (Berraondo et al., 2016; Chen et al., 2017). Cancer immunotherapy has been successfully used in the treatment of many cancers, particularly hematological malignancies and solid tumors (Im and Pavletic, 2017; Nixon et al., 2018). Increasing evidence suggests that immune cells and factors play important roles in the initiation and progression of CRC. Higher proportions of activated CD8^+^ TILs in the early stages of tumor development suggest that immune system surveillance recognizes CRC (Emambux et al., 2018).

Long non-coding RNAs (lncRNAs) are non-translated RNA transcripts and account for 68% of the human transcriptome (Iyer et al., 2015). They are involved in regulating the expression of genes at the epigenetic and transcriptional levels. LncRNAs participate in cell proliferation, differentiation, and apoptosis and play important roles in tumorigenesis and tumor suppression (Ogunwobi et al., 2020). For example, the lncRNAs HEIH, AK023391, and PAGBC can promote CRC tumorigenesis (Huang et al., 2017; Wu et al., 2017; Cui et al., 2018), while the lncRNAs RPPH1, FEZF1-AS1, and u50535 can promote invasion and metastasis of CRC (Bian et al., 2018; Yu et al., 2018; Liang et al., 2019). In this study, we aimed to investigate the relationship between lncRNA gene expression profiles and the immunity characteristics of patients with COAD, and to construct a risk assessment model which can be used to predict the outcome of COAD and identify patients likely to benefit from cancer immunotherapy.

## Materials and Methods

### Data Acquisition and Analysis of Differentially Expressed Genes

We downloaded RNA sequence data and the corresponding clinical information for 497 individuals (41 healthy controls and 456 patients with COAD) from The Cancer Genome Atlas (TCGA) database. The DESeq2 package in R software (3.6.1) was used to identify differentially expressed genes (DEGs) based on the criteria: adj. *P* < 0.05, |logFC| ≥ 1.0, and basemean ≥ 100. A Gene Transfer Format (GTF) file from Ensembl^1^ was used to identify lncRNAs. A list of recognized immune-related genes (IR-Genes) from the ImmPort database^2^ was used to screen immune-related lncRNAs (IR-lncRNAs) using Spearman correlation analysis. David 6.8 was used to carry out KEGG (KEGG: Kyoto Encyclopedia of Genes and Genomes) and GO (Gene Ontology) analysis for those DEGs with the default parameters.

The ESTIMATE package in R software (3.6.1) was used to calculate the immune and stromal scores. The extent of immune cell infiltration was obtained from Tumor Immune Estimation Resource (TIMER) and used to estimate the immune cell infiltrates of patients in the TCGA database^3^.

### Correlation Analysis

Spearman correlation analysis was used to identify the relationship between IR-DEGs and DELs based on the criteria: *P*-value < 0.05 and |R| > 0.5, and to identify the relationship between the risk score and immune infiltration based on the criteria: *P*-value < 0.05 and |R| > 0.

### Overall Survival (OS) Analysis

To conduct OS analysis of IR-DELs, we first grouped the patients with COAD into low- and high-expression groups based on the expression of IR-DELs. Survival, Survminer, and RegParallel packages in R software were used for univariate and multivariate Cox regression analyses. To compute the risk value, we first calculated the cutoff value according to IBM SPSS 22, and then grouped the patients into low- and high-risk groups based on the cutoff value. Survival, Survminer, and RegParallel packages in R software were used for univariate and multivariate Cox regression analyses.

### Risk Assessment Model Construction

We constructed a risk assessment model based on the IR-DELs identified through univariate and multivariate Cox regression analyses. The formula of the risk assessment model was set as follows: risk score = ExpIR-DEL1^∗^βIR-DEL1 + ExpIR-DEL2^∗^βIR-DEL2+……+ ExpIR-DELn^∗^βIR-DELn. Unpaired two-tailed Student’s *t*-test was used to investigate the relationship between the risk assessment model and the clinical characteristics of COAD. Time-dependent receiver operating characteristic (ROC) curves were used to estimate the utility of this model as a prognostic tool for predicting survival status. All statistical analyses were performed in Prism 8.0.1.244.

## Results

### Aberrant Immune and Stromal Scores for COAD

**FIGURE 1:**
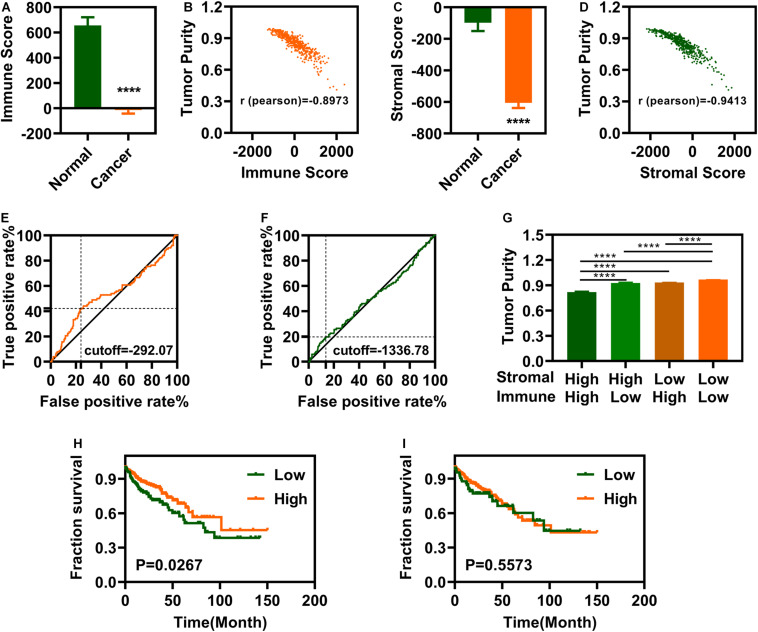
Colon Adenocarcinoma (COAD) is associated with immune and stromal scores. **(A)** Immune scores are significantly associated with COAD. **(B)** Immune scores are significantly correlated with tumor purity. **(C)** Stromal scores are significantly associated with COAD. **(D)** Stromal scores are significantly correlated with tumor purity. **(E,F)** maximum inflection point is the cut-off point for immune scores **(E)** and stromal **(F)**. **(G)** The tumor purity is difference significantly in different groups. **(H)** Patients with COAD with high immune scores group have a longer survival time. **(I)** There is no significantly difference between low stromal scores and high stromal scores groups. ****P < 0.0001.

We analyzed the data of 497 individuals, 41 controls and 456 patients with COAD, downloaded from the TCGA-COAD database. The ESTIMATE package in R software (3.6.1) was used to calculate the immune and stromal scores. The immune and stromal scores for patients with COAD were significantly lower than those for the controls (Figures 1A,C). Immune and stromal scores of COAD showed a significant correlation of both immune and stromal scores with tumor purity predictions (Figures 1B,D). We identified the maximum inflection points of the immune and stromal scores as the cut-off points on the ROC curve. The cutoff values for immune and stromal scores were -292.07 (Figure 1E) and –1336.78 (Figure 1F), respectively. The tumor purity for patients with COAD in different groups was displayed in Figure 1G. After categorizing the patients into low- and high-score groups based on the cutoffs, we performed OS analysis of the immune and stromal scores. Patients with COAD with high immune scores displayed significantly better OS (Figure 1H). However, there were no significant differences in the OS between patients with low and high stromal scores (Figure 1I).

### Identification of Immune-Related Differentially Expressed LncRNAs (IR-DELs)

**FIGURE 2:**
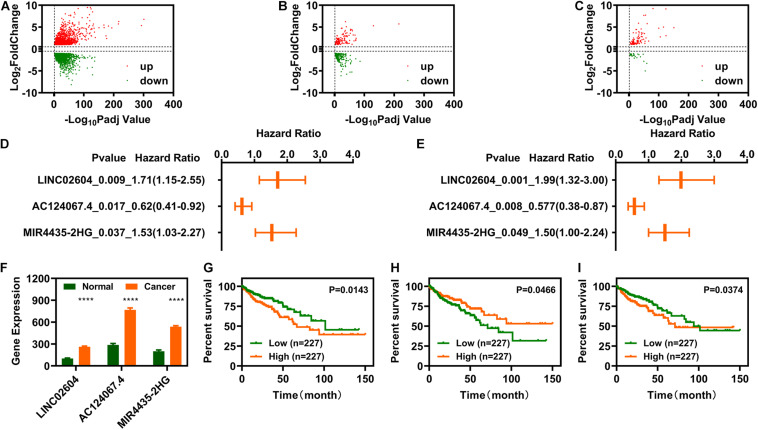
Identification of immune-related differentially expressed long non-coding RNAs (IR-DELs). **(A)** Volcano plot of DEGs for COAD. **(B)** Volcano plot of immune-related DEGs in COAD. **(C)** Volcano plot of immune-related DELs for COAD. **(D)** Heat map of 53 IR-DEGs verified using correlation analysis. **(E)** IR-DELs identified using univariate Cox regression analysis. **(F)** IR-DELs identified through multivariate Cox regression analysis. **(G–I)** Patients with high AC124067.4 expression **(G)**, low LINC02604 expression **(H)**, and low MIR4435-2HG expression **(I)** have a longer survival time. ****P < 0.0001.

Differential expression analysis was carried out using R software’s DEseq2 package. We screened 2977 genes and identified 1502 upregulated and 1475 downregulated genes (Figure 2A). Cross analysis with the recognized IR-genes identified 343 IR-DEGs (118 upregulated and 225 downregulated) (Figure 2B) while cross analysis with GTF annotation data showed 130 DELs (104 upregulated and 26 downregulated) (Figure 2C). To determine the relationship between the 343 IR-DEGs and the 130 DELs, we performed Spearman correlation analysis and obtained 483 pairs of IR-DEGs-DELs, which included 187 IR-DEGs and 53 DELs (Supplementary Table 1). The expression profiles of these 53 DELs was shown in Figure 2D.

Subsequently, we performed univariate Cox regression analysis for the 53 DELs and found that three (AC124067.4, LINC02604, and MIR4435-2HG) were correlated with OS in patients with COAD (Figure 2E). Furthermore, multivariate Cox regression analysis also showed that these three DELs were correlated with OS (Figure 2F). The relationships between AC124067.4, LINC02604, and MIR4435-2HG expression and OS are shown in Figures 2G–I.

### Establishment of the Risk Assessment Model

**FIGURE 3:**
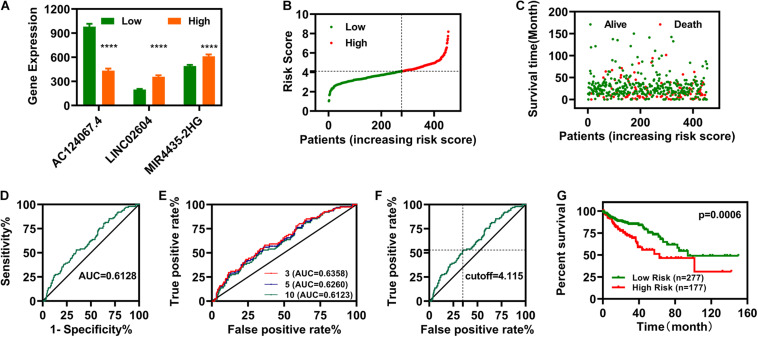
Establishment of a Risk Assessment Model. **(A)** The expression of LINC02604, AC124067.4, and MIR4435-2HG in different groups. **(B,C)** Risk scores **(B)** and survival outcomes **(C)** for each COAD case. **(D)** The ROC curve for the three IR-DELs (AC124067.4, LINC02604, and MIR4435-2HG) models is related to the maximum AUC. **(E)** The 3-, 5-, and 10-year ROC of the optimal model show that all AUC values were over 0.60. **(F)** Risk Scores for 454 patients with COAD; the maximum inflection point is the cut-off point. **(G)** Patients with COAD with low-risk scores have a longer survival time. ****P < 0.0001.

We constructed a risk assessment model using the three DELs (AC124067.4, LINC02604, and MIR4435-2HG). The expression profiles of these three IR-DELs in the normal group and cancer group were shown in Figure 3A. The risk score and survival status of each case are displayed in Figures 3B,C. Next, we calculated the area under the curve (AUC) for each receiver operating characteristic (ROC) curve of the three DELs, generated the curved line, and found that the highest point corresponded to 0.6128 (Figure 3D). To validate the optimality, we further plotted the 3-, 5-, and 10-year ROC curves. All AUC values identified were greater than 0.60 (Figure 3E). The maximum inflection point occurred at a cut-off point of 4.115 (Figure 3F). Using this cutoff point, we re-distinguished the high- and low-risk groups, and then carried out OS analysis. The results indicate that patients with COAD with low-risk scores displayed better OS (Figure 3G). Then, we carried out differential expression analysis for patients with COAD between high-risk group and low-risk group, and found 49 DEGs (34 upregulated and 15 downregulated) (Supplementary Figure 1). We performed KEGG and GO analysis for those 49 DEGs. The results were displayed in Supplementary Table 3.

### Clinical Evaluation Using the Risk Assessment Model

**FIGURE 4:**
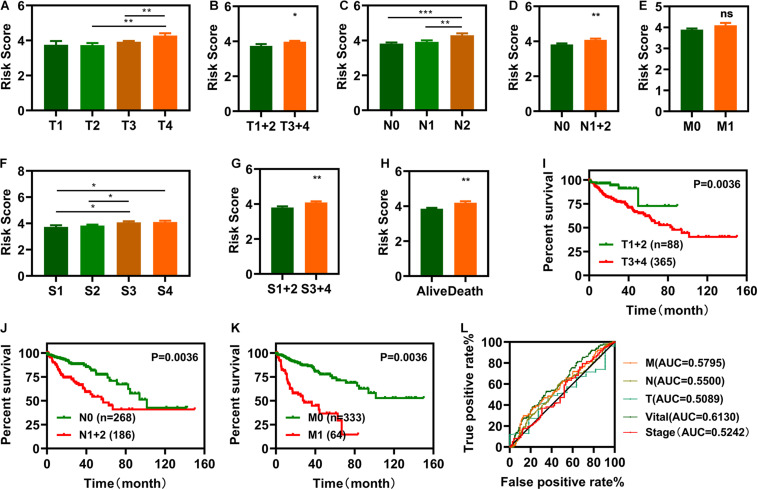
Clinical evaluations with the risk model. **(A–I)** Histograms showing that pathologic tumor (T) **(A,B)**, pathologic node (N) **(C,D)**, pathologic metastasis (M) **(E)**, pathologic stage **(F,G)**, and vital status **(H)** are significantly associated with risk scores. **(I–K)** Patients with COAD in the T1 + 2, N0, and M0 groups have a longer survival time. l, The pathologic N, pathologic M, pathologic T, and pathologic stage ROC of the optimal model showed that all AUC values are lower than those of the vital status. *P < 0.05, **P < 0.01, ***P < 0.001.

To investigate the relationship between risk scores and clinical characteristics, we first computed correlations between risk scores and pathologic TNM (tumor, node and metastasis), pathologic stage, and vital status (Figures 4A–H). Pathologic TNM was previously correlated with OS (Chansky et al., 2009; Wu et al., 2015). In this study, we verified the relationship between pathologic TNM, the pathologic stage, and OS and showed that pathologic TNM was closely associated with OS (Figures 4I–K). We plotted the ROC curves of pathologic TNM, pathologic stage, and vital status, which showed that the AUC values of pathologic TNM and pathologic stage were lower than those of vital status (Figure 4L). These results indicate that the risk model was more accurate at predicting survival status than predicting clinical characteristics. Then, we also analyzed the expression of those the IR-DELs in difference pathologic TNM. The results were displayed in Supplementary Figure 2.

### Estimation of Tumor Immune Infiltration Using the Risk Assessment Model

**FIGURE 5:**
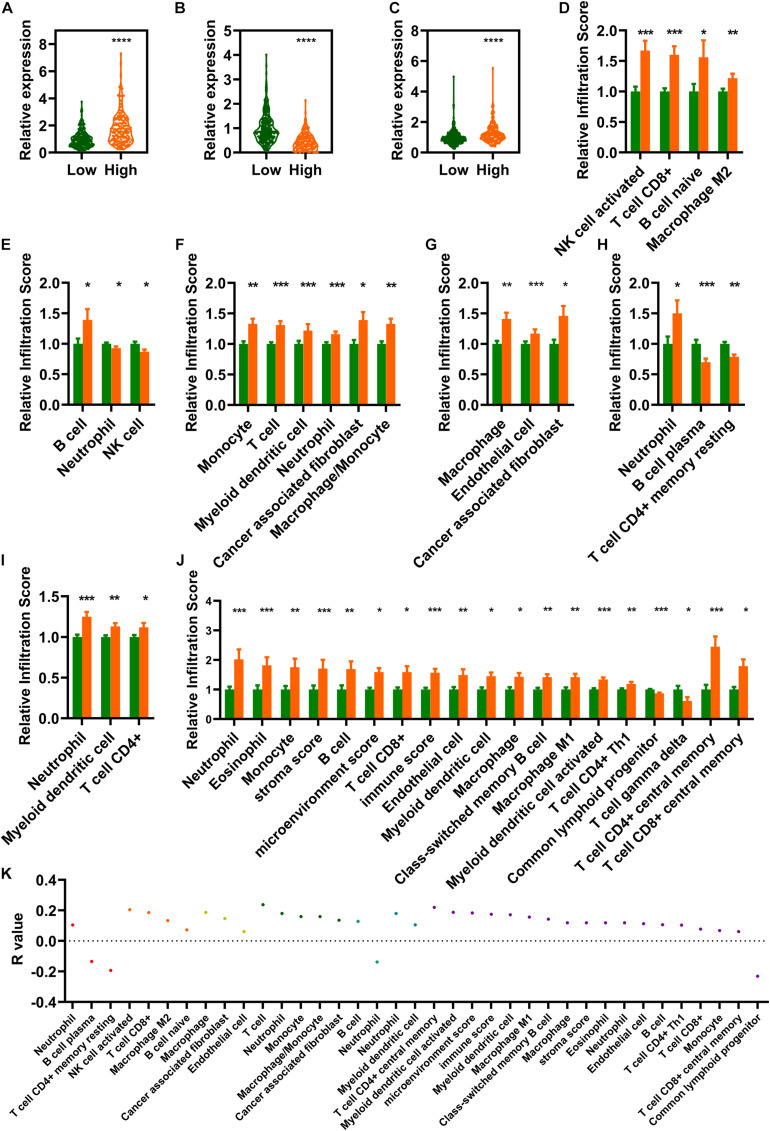
Estimation of tumor immune infiltration using the risk assessment model. **(A–C)** AC124067.4 **(A)**, LINC02604 **(B)**, and MIR4435-2HG **(C)** expression between the low- and high-risk groups. **(D–J)** The infiltrating scores of different tumor immune infiltrations in the low- and high-risk groups are significantly different. **(D)** CIBERSORT-ABS. **(E)** QUANTISEQ. **(F)** MCPCOUNTER. **(G)** EPIC. **(H)** CIBERSORT. **(I)** TIMER. **(J)** XCELL). **(K)** Risk models are significantly correlated with several immune cells and factors (|R| > 0, *p* < 0.05). *P < 0.05, **P < 0.01, ***P < 0.001, ****P < 0.0001.

This study aimed to screen out IR-DELs that could be used to predict COAD outcome by investigating whether the risk score was associated with the three IR-DELs (AC124067.4, LINC02604, and MIR4435-2HG). We found that the expression of one IR-DELs (AC124067.4) and two IR-DELs (LINC02604 and MIR4435-2HG) was negatively and positively correlated with the risk score respectively (Figures 5A–C).

Differential analysis of tumor immune infiltration showed that 73 and 20 immune cells and factors were significantly reduced and increased, respectively, between normal and patients with COAD (Supplementary Table 2). Of these, 41 immune cells and factors were significantly different between the low- and high-risk groups (Figures 5D–J). Spearman correlation analysis of these 41 immune cells and factors showed that 33 and 4 immune cells and factors were positively and negatively correlated with the risk score value respectively (Figure 5K).

## Discussion

CRC is a common and aggressive cancer associated with high mortality. Although overall mortality rates continue to decline due to improvements in diagnosis and treatment, the survival rate for advanced disease remains low and approximately 50% of patients relapse even after additional treatment with neoadjuvant therapy (Ogura et al., 2019). Cancer immunotherapy is a new alternative for cancer treatment that overcomes the non-specific problems associated with radiotherapy and chemotherapy (Johdi and Sukor, 2020). Hence, it is important to find a suitable immunity-related prognostic biomarker for CRC and to identify patients who can benefit from anti-tumor immunotherapy.

In this study, we identified three IR-DEGs (AC124067.4, LINC02604, and MIR4435-2HG) as possible prognostic biomarkers. AC124067.4, also known as RP11-150O12.3, is located on the shorter arm of chromosome 8 (8p). AC124067.4 was previously demonstrated to be an independent predictor of gastric cancer prognosis (Ren et al., 2016) and its expression was associated with survival in patients with CRC (Poursheikhani et al., 2020). Our study found that the expression of AC124067.4 was significantly upregulated, and its expression level was also associated with the OS rate in patients with COAD. Our findings are consistent with previous reports, reinforcing the possibility of using AC124067.4 as a prognostic biomarker for COAD. LINC02604, also known as LncHERG, is located on the longer arm of chromosome 7 (7q). Shi et al. (2017) found that LINC02604 knockdown inhibited glioblastoma cell proliferation, migration, and invasion *in vitro* and *in vivo*. These inhibitory effects were achieved by regulating miR-940 (Shi et al., 2017). In addition, they also found that patients with glioblastoma with high LINC02604 expression had poor prognosis and low survival (Shi et al., 2017). In the present study, we found that LINC02604 expression was significantly increased in patients with COAD, and they showed poor OS rate, consistent with a previous study (Shi et al., 2017). MIR4435-2HG is located on human chromosome 2q13. Previous studies correlated MIR4435-2HG with several cancers, including gastric cancer, hepatocellular cancer, ovarian cancer, and CRC (Freitas-Andrade et al., 2019; Gong et al., 2019; Kong et al., 2019; Dong et al., 2020; Shen and Zhou, 2020; Zhu et al., 2020). Freitas-Andrade et al. (2019) found that MIR4435-2HG expression was significantly increased in gastric cancer and promoted the growth and metastasis of gastric cancer by activating the Wnt signaling pathway. Kong et al. (2019) found that MIR4435-2HG expression was significantly increased in hepatocellular cancer and could promote proliferation by upregulating miRNA-487a. MIR4435-2HG has been identified as an early diagnostic biomarker for ovarian cancer (Gong et al., 2019) and gastric cancer (Wang et al., 2019). Ke et al. (2017) found that MIR4435-2HG expression was increased in colorectal cancer, and patients with high MIR4435-2HG expression displayed poorer progression-free survival and overall survival. Our results further indicate that MIR4435-2HG can be used as a prognostic biomarker to predict COAD outcome.

Till now, there are many risk assessment models for COAD based on differential expression analysis, including differentially expressed genes, differentially expressed lncRNA, differentially expressed microRNA. Li et al. (2020) found that a risk signature constructed by 10 genes (*CEBPB*, *CXCL9*, *IRF8*, *ITGB1*, *LAG3*, *MCFD2*, *PSMD11*, *RNASE7*, *SPARC*, and *TAP2*) displayed an accuracy of predictions of survival (AUC = 0.6763/0.6465). Miao et al. (2020) constructed a prognosis model by using 12 immune genes, including *SLC10A2*, *CXCL3*, *NOX4*, *FABP4*, *ADIPOQ*, *IGKV1*-*33*, *IGLV6*-*57*, *INHBA*, *UCN*, *VIP*, *NGFR*, and *TRDC*. The OS was significantly lower in the high-risk group than in the low-risk group. The 1-, 3-, and 5-year AUC value of ROC was 0.625, 0.646, and 0.713, respectively (Miao et al., 2020). Dai et al. (2018) also constructed a risk signature based on 16 genes (*ATOH1*, *CDC6*, *CXCL10*, *EGR3*, *GALNT4*, *GZMB*, *HSD17B2*, *IFI6*, *INHBB*, *KLK11*, *OSER1*-*AS1*, *PLAT*, *PTPRR*, *RAB15*, *SPAG1*, and *SPINK1*) and 2 lncRNAs (*PRKAG2-AS1* and *SNHG17*). The 1-, 3-, and 5-year AUC value of ROC in the external validation set was 0.733, 0.667, and 0.673, respectively (Dai et al., 2018). In the present study, we constructed a risk assessment model using three IR-DELs. The AUC value of ROC was 0.6128 which was comparable with the risk model constructed by Li et al. and Miao et al., lower than the risk model constructed by Dai et al. (2018) Comparatively, there were fewer DEGs or DELs used in our present risk assessment model. In conclusion, we identified three IR-DELs that can be used as prognostic biomarkers for COAD and constructed a risk prediction model. However, whether the risk assessment models constructed by previous studies or our present investigation now are feasible in clinical prediction needs to be further verified.

## Data Availability Statement

The original contributions presented in the study are included in the article/Supplementary Material, further inquiries can be directed to the corresponding author/s.

## Author Contributions

MH and X-LX conceived and designed the experiments. X-LX performed the analysis. Z-YY, TZ, CX, and CW helped to analyze the data. Y-WL and X-LX wrote the manuscript. All authors contributed to the article and approved the submitted version.

## Conflict of Interest

The authors declare that the research was conducted in the absence of any commercial or financial relationships that could be construed as a potential conflict of interest.

## Footnotes

1 http://asia.ensembl.org

2 http://www.immport.org

3 https://cistrome.shinyapps.io/timer/
