# Supplementary material for: Immune-Related Gene Expression Analysis Revealed Three lncRNAs as Prognostic Factors for Colon Cancer
Source: Front Genet. 2021 Jul 9;12:690053. doi: 10.3389/fgene.2021.690053 (PMC8299306; doi:10.3389/fgene.2021.690053)
Supplement: Supplementary file 1 [file Data_Sheet_1.docx]

**Immune-related Gene Expression Analysis Revealed three lncRNAs as Prognostic factors for Colon Cancer**

Xiao-Liang Xing^1^, Ti Zhang^1^, Zhi-Yong Yao^1^, Chaoqun Xing^1^, Chunxiao Wang^1^, Yuan-Wu Liu^2^, Minjiang Huang^1^*.

^1^Hunan University of Medicine, Huaihua 418000, Hunan, P. R. China.

^2^Beijing Advanced Innovation Center for Food Nutrition and Human Health, China Agricultural University, 100193, Beijing, China.

*Correspondence: Minjiang Huang, whmj530@163.com.

**Supplementary information: 3 tables and 2 figures**

**Supplementary table 1 The screened pairs of IR-DEGs and DELs by Spearman correlation analysis**

| **IR-DEGs** | **DELs** | **P** | **R** | **IR-DEGs** | **DELs** | **P** | **R** |
| --- | --- | --- | --- | --- | --- | --- | --- |
| FGF7 | MAGI2-AS3 | 0.000 | 0.87 | LTB4R | AL691482.3 | 0.000 | 0.55 |
| AKT3 | MAGI2-AS3 | 0.000 | 0.86 | FCGR2B | AC104083.1 | 0.000 | 0.55 |
| AKT3 | MIR100HG | 0.000 | 0.84 | IL16 | MAGI2-AS3 | 0.000 | 0.55 |
| FGF7 | MIR100HG | 0.000 | 0.84 | IGKV4-1 | MIR22HG | 0.000 | 0.55 |
| NR3C1 | MAGI2-AS3 | 0.000 | 0.83 | CCL28 | LINC01133 | 0.000 | 0.55 |
| PTGER3 | MIR100HG | 0.000 | 0.83 | IGHV3-21 | MIR22HG | 0.000 | 0.55 |
| AKT3 | AC104083.1 | 0.000 | 0.82 | IGLV2-14 | MIR22HG | 0.000 | 0.55 |
| COLEC12 | MAGI2-AS3 | 0.000 | 0.81 | CMKLR1 | CARMN | 0.000 | 0.55 |
| FGF7 | AC104083.1 | 0.000 | 0.80 | LTB4R | ASMTL-AS1 | 0.000 | 0.55 |
| PTGER3 | MAGI2-AS3 | 0.000 | 0.80 | CMTM8 | SNHG11 | 0.000 | 0.55 |
| TEK | MAGI2-AS3 | 0.000 | 0.79 | IL10RA | CARMN | 0.000 | 0.55 |
| PTGER3 | AC104083.1 | 0.000 | 0.79 | LEPR | MIR100HG | 0.000 | 0.55 |
| SLIT2 | MAGI2-AS3 | 0.000 | 0.78 | ANOS1 | AC104083.1 | 0.000 | 0.55 |
| COLEC12 | MIR100HG | 0.000 | 0.78 | S1PR1 | FENDRR | 0.000 | 0.54 |
| SLIT2 | MIR100HG | 0.000 | 0.77 | IGHA2 | MIR22HG | 0.000 | 0.54 |
| ANGPTL1 | MAGI2-AS3 | 0.000 | 0.77 | CCL28 | AC025580.1 | 0.000 | 0.54 |
| A2M | MAGI2-AS3 | 0.000 | 0.77 | IL10RA | MIR100HG | 0.000 | 0.54 |
| ANGPTL1 | MIR100HG | 0.000 | 0.77 | CHP2 | AC254629.1 | 0.000 | 0.54 |
| S1PR1 | MAGI2-AS3 | 0.000 | 0.77 | CXCL12 | MBNL1-AS1 | 0.000 | 0.54 |
| PDGFRA | MAGI2-AS3 | 0.000 | 0.76 | LCN12 | AL117382.1 | 0.000 | 0.54 |
| ANGPTL1 | CARMN | 0.000 | 0.75 | IGHV3-15 | MIR22HG | 0.000 | 0.54 |
| EDNRB | FENDRR | 0.000 | 0.75 | IGLV3-1 | MIR22HG | 0.000 | 0.54 |
| NRP2 | MAGI2-AS3 | 0.000 | 0.75 | CSF2RB | CARMN | 0.000 | 0.54 |
| AKT3 | CARMN | 0.000 | 0.74 | CCL20 | MIR3142HG | 0.000 | 0.54 |
| PDGFRA | FENDRR | 0.000 | 0.74 | S100B | FENDRR | 0.000 | 0.54 |
| PYY | CDKN2B-AS1 | 0.000 | 0.74 | IGKV1-16 | MIR22HG | 0.000 | 0.54 |
| NRP2 | MIR100HG | 0.000 | 0.74 | PIK3CG | MIR22HG | 0.000 | 0.54 |
| LIFR | MAGI2-AS3 | 0.000 | 0.73 | VEGFA | RUSC1-AS1 | 0.000 | 0.54 |
| NR3C1 | MIR100HG | 0.000 | 0.73 | IGLV4-69 | MIR22HG | 0.000 | 0.54 |
| OGN | MAGI2-AS3 | 0.000 | 0.73 | BTK | AC104083.1 | 0.000 | 0.54 |
| IL6ST | MAGI2-AS3 | 0.000 | 0.73 | ADA2 | MIR100HG | 0.000 | 0.54 |
| GREM2 | FENDRR | 0.000 | 0.73 | IGLV7-46 | CDKN2B-AS1 | 0.000 | 0.54 |
| NPR3 | MIR100HG | 0.000 | 0.72 | FABP6 | LINC01234 | 0.000 | 0.54 |
| PTGER3 | CARMN | 0.000 | 0.72 | LTB4R | SLC9A3-AS1 | 0.000 | 0.54 |
| NRP2 | AC104083.1 | 0.000 | 0.71 | PIK3R5 | MIR100HG | 0.000 | 0.54 |
| NR3C1 | AC104083.1 | 0.000 | 0.71 | TDGF1 | SNHG11 | 0.000 | 0.54 |
| SLC22A17 | CARMN | 0.000 | 0.71 | GUCA2A | LINC02747 | 0.000 | 0.54 |
| FGFR1 | MIR100HG | 0.000 | 0.71 | SEMA3G | MBNL1-AS1 | 0.000 | 0.54 |
| FGFR1 | MAGI2-AS3 | 0.000 | 0.71 | CD79B | MIR22HG | 0.000 | 0.54 |
| TEK | AC104083.1 | 0.000 | 0.70 | CD48 | MIR22HG | 0.000 | 0.54 |
| CXCL12 | MAGI2-AS3 | 0.000 | 0.70 | IGHA1 | CDKN2B-AS1 | 0.000 | 0.54 |
| SLIT2 | AC104083.1 | 0.000 | 0.70 | ACKR1 | AC104083.1 | 0.000 | 0.54 |
| OGN | MIR100HG | 0.000 | 0.70 | INHBA | AC104083.1 | 0.000 | 0.54 |
| A2M | MIR100HG | 0.000 | 0.70 | BTK | MIR22HG | 0.000 | 0.54 |
| GUCA2A | CDKN2B-AS1 | 0.000 | 0.70 | SOS2 | MBNL1-AS1 | 0.000 | 0.54 |
| GCG | CDKN2B-AS1 | 0.000 | 0.70 | CHP2 | CDKN2B-AS1 | 0.000 | 0.54 |
| PTN | FENDRR | 0.000 | 0.70 | FGF2 | CARMN | 0.000 | 0.54 |
| ANGPTL1 | AC104083.1 | 0.000 | 0.70 | IGLV7-43 | MIR22HG | 0.000 | 0.54 |
| FGF2 | MAGI2-AS3 | 0.000 | 0.70 | IGKV3-20 | MIR22HG | 0.000 | 0.54 |
| COLEC12 | AC104083.1 | 0.000 | 0.70 | TLR1 | AC104083.1 | 0.000 | 0.54 |
| S1PR1 | MIR100HG | 0.000 | 0.70 | IGHV3-72 | MIR22HG | 0.000 | 0.53 |
| SLC22A17 | MIR100HG | 0.000 | 0.69 | PTGER3 | MBNL1-AS1 | 0.000 | 0.53 |
| AQP9 | PELATON | 0.000 | 0.69 | IGHA2 | FENDRR | 0.000 | 0.53 |
| TEK | MIR100HG | 0.000 | 0.69 | IGHV3-72 | CDKN2B-AS1 | 0.000 | 0.53 |
| COLEC12 | CARMN | 0.000 | 0.69 | EREG | AL117382.1 | 0.000 | 0.53 |
| BTK | MAGI2-AS3 | 0.000 | 0.69 | IGLV1-40 | MIR22HG | 0.000 | 0.53 |
| A2M | AC104083.1 | 0.000 | 0.69 | EREG | AC078993.1 | 0.000 | 0.53 |
| TNFSF4 | MIR100HG | 0.000 | 0.68 | SEMA3G | CARMN | 0.000 | 0.53 |
| LCN12 | AL390719.2 | 0.000 | 0.68 | IL1RN | PELATON | 0.000 | 0.53 |
| FGF7 | CARMN | 0.000 | 0.68 | IGHV3-11 | MIR22HG | 0.000 | 0.53 |
| FCGR2B | MIR100HG | 0.000 | 0.68 | PTPRC | MIR100HG | 0.000 | 0.53 |
| NPR3 | MAGI2-AS3 | 0.000 | 0.68 | RBP5 | FENDRR | 0.000 | 0.53 |
| SLC11A1 | PELATON | 0.000 | 0.68 | BTK | CARMN | 0.000 | 0.53 |
| CHGA | CDKN2B-AS1 | 0.000 | 0.68 | PIK3CG | MIR100HG | 0.000 | 0.53 |
| LCN12 | MHENCR | 0.000 | 0.67 | CMTM8 | MNX1-AS1 | 0.000 | 0.53 |
| S1PR1 | AC104083.1 | 0.000 | 0.67 | FGFR4 | SNHG17 | 0.000 | 0.53 |
| SLC22A17 | MAGI2-AS3 | 0.000 | 0.67 | PI15 | MIR100HG | 0.000 | 0.53 |
| FCGR2B | MAGI2-AS3 | 0.000 | 0.67 | FABP6 | SNHG11 | 0.000 | 0.53 |
| LCN12 | AL355987.4 | 0.000 | 0.67 | CD1D | FENDRR | 0.000 | 0.53 |
| OGN | AC104083.1 | 0.000 | 0.67 | CHP2 | PP7080 | 0.000 | 0.53 |
| A2M | CARMN | 0.000 | 0.67 | IGLV1-47 | MIR22HG | 0.000 | 0.53 |
| LCN12 | ASMTL-AS1 | 0.000 | 0.67 | CHGB | FENDRR | 0.000 | 0.53 |
| PTN | MAGI2-AS3 | 0.000 | 0.67 | IGHV1-46 | MIR22HG | 0.000 | 0.53 |
| PDF | SNHG25 | 0.000 | 0.67 | TAFA5 | AC104083.1 | 0.000 | 0.53 |
| CCL21 | MIR100HG | 0.000 | 0.67 | IGLV2-23 | MIR22HG | 0.000 | 0.53 |
| LCN12 | TMEM147-AS1 | 0.000 | 0.67 | EDN3 | CDKN2B-AS1 | 0.000 | 0.53 |
| SLIT2 | CARMN | 0.000 | 0.67 | CCL13 | MIR22HG | 0.000 | 0.53 |
| PDGFRA | AC104083.1 | 0.000 | 0.67 | IGKC | MIR22HG | 0.000 | 0.53 |
| TLR1 | MAGI2-AS3 | 0.000 | 0.66 | IGKV3-11 | MIR22HG | 0.000 | 0.53 |
| CCL21 | MAGI2-AS3 | 0.000 | 0.66 | NGFR | MIR100HG | 0.000 | 0.53 |
| TNFSF4 | AC104083.1 | 0.000 | 0.66 | VIP | AC104083.1 | 0.000 | 0.53 |
| TEK | CARMN | 0.000 | 0.66 | IGLV7-46 | MIR22HG | 0.000 | 0.53 |
| FGFR1 | CARMN | 0.000 | 0.66 | CSF2RB | MIR100HG | 0.000 | 0.53 |
| A2M | FENDRR | 0.000 | 0.66 | IL6R | CDKN2B-AS1 | 0.000 | 0.53 |
| IL6ST | AC104083.1 | 0.000 | 0.66 | IGLV2-11 | MIR22HG | 0.000 | 0.53 |
| ANGPTL1 | MBNL1-AS1 | 0.000 | 0.66 | SEMA6D | FENDRR | 0.000 | 0.53 |
| FGF2 | MIR100HG | 0.000 | 0.66 | CD79A | MIR22HG | 0.000 | 0.53 |
| SCG2 | MAGI2-AS3 | 0.000 | 0.66 | IGHV3-23 | MIR22HG | 0.000 | 0.53 |
| NRP2 | CARMN | 0.000 | 0.65 | IGHV5-51 | MIR22HG | 0.000 | 0.52 |
| PDGFRA | MIR100HG | 0.000 | 0.65 | CD4 | MIR100HG | 0.000 | 0.52 |
| CCL11 | FENDRR | 0.000 | 0.65 | IGHV1-18 | MIR22HG | 0.000 | 0.52 |
| LIFR | MIR100HG | 0.000 | 0.65 | IGKV1-8 | MIR22HG | 0.000 | 0.52 |
| FGF2 | AC104083.1 | 0.000 | 0.65 | INHBA | PELATON | 0.000 | 0.52 |
| SCG2 | MIR100HG | 0.000 | 0.65 | VEGFA | AC020916.1 | 0.000 | 0.52 |
| FGFR1 | AC104083.1 | 0.000 | 0.65 | PTGER4 | AC025580.1 | 0.000 | 0.52 |
| TPM2 | MIR100HG | 0.000 | 0.65 | PTN | MBNL1-AS1 | 0.000 | 0.52 |
| BID | SNHG25 | 0.000 | 0.65 | CCL13 | CDKN2B-AS1 | 0.000 | 0.52 |
| FABP4 | MIR100HG | 0.000 | 0.65 | INSL5 | CDKN2B-AS1 | 0.000 | 0.52 |
| LIFR | FENDRR | 0.000 | 0.64 | FABP4 | AC104083.1 | 0.000 | 0.52 |
| NPR1 | CARMN | 0.000 | 0.64 | IGHV3-66 | MIR22HG | 0.000 | 0.52 |
| PTPRC | MAGI2-AS3 | 0.000 | 0.64 | COLEC12 | MBNL1-AS1 | 0.000 | 0.52 |
| NR3C1 | CARMN | 0.000 | 0.64 | IGLV1-44 | MIR22HG | 0.000 | 0.52 |
| BMP3 | FENDRR | 0.000 | 0.64 | PLXNA3 | AL691482.3 | 0.000 | 0.52 |
| OLR1 | PELATON | 0.000 | 0.64 | CCL11 | MAGI2-AS3 | 0.000 | 0.52 |
| OGN | CARMN | 0.000 | 0.64 | FGF2 | MBNL1-AS1 | 0.000 | 0.52 |
| S1PR1 | CARMN | 0.000 | 0.63 | PLCG2 | MAGI2-AS3 | 0.000 | 0.52 |
| PIK3CG | MAGI2-AS3 | 0.000 | 0.63 | VEGFA | ASMTL-AS1 | 0.000 | 0.52 |
| ADA2 | MAGI2-AS3 | 0.000 | 0.63 | LCN12 | LINC01315 | 0.000 | 0.52 |
| FABP4 | MAGI2-AS3 | 0.000 | 0.63 | IGKV1-27 | MIR22HG | 0.000 | 0.52 |
| CMKLR1 | MAGI2-AS3 | 0.000 | 0.63 | VEGFA | C6orf223 | 0.000 | 0.52 |
| TNFSF4 | MAGI2-AS3 | 0.000 | 0.63 | IL6ST | CARMN | 0.000 | 0.52 |
| PRKCB | MAGI2-AS3 | 0.000 | 0.63 | IGLV5-45 | MIR22HG | 0.000 | 0.52 |
| SLIT2 | MBNL1-AS1 | 0.000 | 0.63 | PLCG2 | CARMN | 0.000 | 0.52 |
| CSF2RB | MAGI2-AS3 | 0.000 | 0.63 | CSRP1 | MIR100HG | 0.000 | 0.52 |
| DES | CARMN | 0.000 | 0.63 | IGLV4-60 | MIR22HG | 0.000 | 0.52 |
| PLXNA3 | ASMTL-AS1 | 0.000 | 0.62 | IGHM | MIR22HG | 0.000 | 0.52 |
| FABP4 | CARMN | 0.000 | 0.62 | NR3C1 | FENDRR | 0.000 | 0.52 |
| BMP3 | CDKN2B-AS1 | 0.000 | 0.62 | IGLV7-43 | CDKN2B-AS1 | 0.000 | 0.52 |
| SCG2 | CARMN | 0.000 | 0.62 | TAFA5 | CASC15 | 0.000 | 0.52 |
| NPR3 | AC104083.1 | 0.000 | 0.62 | INHBA | MIR100HG | 0.000 | 0.52 |
| PIK3R5 | MAGI2-AS3 | 0.000 | 0.62 | PLCG2 | MIR22HG | 0.000 | 0.52 |
| CCL21 | CARMN | 0.000 | 0.62 | PLAU | MIR4435-2HG | 0.000 | 0.52 |
| SEMA3G | MAGI2-AS3 | 0.000 | 0.62 | CMKLR1 | AC104083.1 | 0.000 | 0.52 |
| TPM2 | CARMN | 0.000 | 0.62 | IGLV2-18 | CDKN2B-AS1 | 0.000 | 0.52 |
| PDGFD | FENDRR | 0.000 | 0.61 | SAA2 | MIR3142HG | 0.000 | 0.52 |
| BTK | MIR100HG | 0.000 | 0.61 | IGKV2-24 | MIR22HG | 0.000 | 0.52 |
| ACKR1 | MAGI2-AS3 | 0.000 | 0.61 | CD209 | MIR22HG | 0.000 | 0.52 |
| LIFR | AC104083.1 | 0.000 | 0.61 | PLXNA2 | AP002761.4 | 0.000 | 0.52 |
| IL10RA | MAGI2-AS3 | 0.000 | 0.61 | IGHV3-73 | MIR22HG | 0.000 | 0.52 |
| CHP2 | LINC02747 | 0.000 | 0.61 | IGLV3-25 | MIR22HG | 0.000 | 0.52 |
| TDGF1 | TUSC8 | 0.000 | 0.61 | IGHV4-34 | MIR22HG | 0.000 | 0.52 |
| MASP1 | FENDRR | 0.000 | 0.61 | PIK3CD | MIR22HG | 0.000 | 0.52 |
| PTN | MIR100HG | 0.000 | 0.61 | CD1D | CDKN2B-AS1 | 0.000 | 0.52 |
| IL6ST | MIR100HG | 0.000 | 0.61 | IGKV1-9 | MIR22HG | 0.000 | 0.52 |
| CCL21 | AC104083.1 | 0.000 | 0.61 | PTPRC | AC104083.1 | 0.000 | 0.52 |
| ACKR1 | MIR100HG | 0.000 | 0.61 | PLXNA3 | TMEM147-AS1 | 0.000 | 0.52 |
| LCN12 | SNHG20 | 0.000 | 0.61 | IGLV6-57 | MIR22HG | 0.000 | 0.52 |
| CXCL12 | CARMN | 0.000 | 0.60 | CDK4 | MNX1-AS1 | 0.000 | 0.51 |
| CXCL12 | MIR100HG | 0.000 | 0.60 | CXCL3 | MIR3142HG | 0.000 | 0.51 |
| LCN12 | SNHG11 | 0.000 | 0.60 | IGHA2 | AC025580.1 | 0.000 | 0.51 |
| LCN12 | LINC02604 | 0.000 | 0.60 | IGKV1-5 | MIR22HG | 0.000 | 0.51 |
| CXCL12 | AC104083.1 | 0.000 | 0.60 | SLC11A1 | AGAP2-AS1 | 0.000 | 0.51 |
| TEK | FENDRR | 0.000 | 0.60 | CMTM8 | MHENCR | 0.000 | 0.51 |
| CCL3 | PELATON | 0.000 | 0.60 | OLR1 | MIR100HG | 0.000 | 0.51 |
| PTN | AC104083.1 | 0.000 | 0.59 | IGLV2-18 | MIR22HG | 0.000 | 0.51 |
| SCG2 | AC104083.1 | 0.000 | 0.59 | ADA2 | AC104083.1 | 0.000 | 0.51 |
| LEPR | MAGI2-AS3 | 0.000 | 0.59 | GHR | MAGI2-AS3 | 0.000 | 0.51 |
| ACKR1 | CARMN | 0.000 | 0.59 | RSAD2 | MIR22HG | 0.000 | 0.51 |
| CD4 | MAGI2-AS3 | 0.000 | 0.59 | MIF | SNHG25 | 0.000 | 0.51 |
| LCN12 | TSPOAP1-AS1 | 0.000 | 0.59 | IGKV3-15 | MIR22HG | 0.000 | 0.51 |
| NPR3 | CARMN | 0.000 | 0.59 | IGLV2-8 | MIR22HG | 0.000 | 0.51 |
| LTB4R | AL390719.2 | 0.000 | 0.59 | IGLV1-51 | MIR22HG | 0.000 | 0.51 |
| DES | MIR100HG | 0.000 | 0.59 | IGHV4-59 | MIR22HG | 0.000 | 0.51 |
| TNFSF12 | MIR100HG | 0.000 | 0.59 | CXCL2 | MIR3142HG | 0.000 | 0.51 |
| PI15 | MAGI2-AS3 | 0.000 | 0.59 | EDNRA | MAGI2-AS3 | 0.000 | 0.51 |
| LIFR | CARMN | 0.000 | 0.59 | INHBA | MAGI2-AS3 | 0.000 | 0.51 |
| SLC22A17 | FENDRR | 0.000 | 0.59 | IGKV1-17 | MIR22HG | 0.000 | 0.51 |
| CSF1R | MAGI2-AS3 | 0.000 | 0.59 | SECTM1 | MIR22HG | 0.000 | 0.51 |
| AKT3 | MBNL1-AS1 | 0.000 | 0.59 | LIF | AC020916.1 | 0.000 | 0.51 |
| SPP1 | PELATON | 0.000 | 0.59 | CHGA | FENDRR | 0.000 | 0.51 |
| VIPR1 | AC254629.1 | 0.000 | 0.59 | CMTM8 | LINC01315 | 0.000 | 0.51 |
| MASP1 | MIR100HG | 0.000 | 0.59 | VEGFA | AL691482.3 | 0.000 | 0.51 |
| LTB4R | AC087741.1 | 0.000 | 0.59 | IGHV3-48 | MIR22HG | 0.000 | 0.51 |
| OGN | MBNL1-AS1 | 0.000 | 0.59 | HMOX1 | MIR22HG | 0.000 | 0.51 |
| TDGF1 | AL117382.1 | 0.000 | 0.58 | DES | AC104083.1 | 0.000 | 0.51 |
| NPR1 | MAGI2-AS3 | 0.000 | 0.58 | IGHV3-53 | MIR22HG | 0.000 | 0.51 |
| FCGR2B | CARMN | 0.000 | 0.58 | BIRC5 | AC092718.4 | 0.000 | 0.51 |
| PLXNA3 | LINC02604 | 0.000 | 0.58 | CSRP1 | MBNL1-AS1 | 0.000 | 0.51 |
| EDNRA | CASC15 | 0.000 | 0.58 | IGKV3D-20 | MIR22HG | 0.000 | 0.51 |
| VIP | MIR100HG | 0.000 | 0.58 | PDF | VPS9D1-AS1 | 0.000 | 0.51 |
| IGHA2 | CDKN2B-AS1 | 0.000 | 0.58 | IGHV3-74 | CDKN2B-AS1 | 0.000 | 0.51 |
| RBP5 | CARMN | 0.000 | 0.58 | CSF1 | MIR100HG | 0.000 | 0.51 |
| TPM2 | AC104083.1 | 0.000 | 0.58 | TDGF1 | LINC01315 | 0.000 | 0.51 |
| LTB4R | MMP25-AS1 | 0.000 | 0.58 | IGHV3-33 | MIR22HG | 0.000 | 0.51 |
| TLR1 | MIR100HG | 0.000 | 0.58 | NRP2 | MBNL1-AS1 | 0.000 | 0.51 |
| OSM | PELATON | 0.000 | 0.58 | S1PR1 | MBNL1-AS1 | 0.000 | 0.51 |
| GREM2 | MIR100HG | 0.000 | 0.58 | CD22 | MIR22HG | 0.000 | 0.51 |
| NGFR | CARMN | 0.000 | 0.58 | LCN12 | SNHG15 | 0.000 | 0.51 |
| MASP1 | CARMN | 0.000 | 0.57 | ACVRL1 | AC025580.1 | 0.000 | 0.51 |
| SLC22A17 | AC104083.1 | 0.000 | 0.57 | CD48 | MAGI2-AS3 | 0.000 | 0.51 |
| CSF1 | MAGI2-AS3 | 0.000 | 0.57 | TDGF1 | AC078993.1 | 0.000 | 0.51 |
| PTN | CARMN | 0.000 | 0.57 | BID | LINC01315 | 0.000 | 0.51 |
| IL6ST | MBNL1-AS1 | 0.000 | 0.57 | CMTM8 | MCF2L-AS1 | 0.000 | 0.51 |
| PDGFD | MAGI2-AS3 | 0.000 | 0.57 | LEPR | AC104083.1 | 0.000 | 0.51 |
| PDGFRA | CARMN | 0.000 | 0.57 | CDK4 | SNHG15 | 0.000 | 0.51 |
| LCN12 | AC087741.1 | 0.000 | 0.57 | EDNRA | MIR100HG | 0.000 | 0.51 |
| TEK | MBNL1-AS1 | 0.000 | 0.57 | FABP4 | MBNL1-AS1 | 0.000 | 0.51 |
| TDGF1 | LINC02418 | 0.000 | 0.57 | MASP1 | MBNL1-AS1 | 0.000 | 0.50 |
| LCN12 | AL691482.3 | 0.000 | 0.57 | FGFR4 | AC104958.2 | 0.000 | 0.50 |
| PLAU | PELATON | 0.000 | 0.57 | IGHV4-28 | MIR22HG | 0.000 | 0.50 |
| TPM2 | MAGI2-AS3 | 0.000 | 0.57 | DES | FENDRR | 0.000 | 0.50 |
| GREM2 | CARMN | 0.000 | 0.57 | IGKV2-24 | CDKN2B-AS1 | 0.000 | 0.50 |
| LIFR | MBNL1-AS1 | 0.000 | 0.57 | IGLC2 | MIR22HG | 0.000 | 0.50 |
| ACKR1 | FENDRR | 0.000 | 0.57 | PTGDS | FENDRR | 0.000 | 0.50 |
| VIP | FENDRR | 0.000 | 0.57 | IGKV1-6 | MIR22HG | 0.000 | 0.50 |
| LCN12 | SLC9A3-AS1 | 0.000 | 0.57 | FGFR4 | AL117382.1 | 0.000 | 0.50 |
| NMB | SNHG25 | 0.000 | 0.57 | GREM2 | AC104083.1 | 0.000 | 0.50 |
| IGHA1 | MIR22HG | 0.000 | 0.56 | IGHV3-74 | FENDRR | 0.000 | 0.50 |
| LCN2 | MIR3142HG | 0.000 | 0.56 | INHBA | CASC15 | 0.000 | 0.50 |
| AHNAK | MBNL1-AS1 | 0.000 | 0.56 | FCGR2B | MIR22HG | 0.000 | 0.50 |
| DES | MBNL1-AS1 | 0.000 | 0.56 | ANOS1 | MIR100HG | 0.000 | 0.50 |
| LCN12 | SNHG17 | 0.000 | 0.56 | IGHV3-49 | MIR22HG | 0.000 | 0.50 |
| TNFSF12 | MAGI2-AS3 | 0.000 | 0.56 | CXCL12 | FENDRR | 0.000 | 0.50 |
| NR3C1 | MBNL1-AS1 | 0.000 | 0.56 | IGKV1-16 | CDKN2B-AS1 | 0.000 | 0.50 |
| IL10RA | MIR22HG | 0.000 | 0.56 | CD1D | MIR22HG | 0.000 | 0.50 |
| EDNRA | AC104083.1 | 0.000 | 0.56 | INHBA | MIR4435-2HG | 0.000 | 0.50 |
| MASP1 | AC104083.1 | 0.000 | 0.56 | IGHV4-39 | MIR22HG | 0.000 | 0.50 |
| LTB4R | RUSC1-AS1 | 0.000 | 0.56 | PRKCB | AC104083.1 | 0.000 | 0.50 |
| IL16 | MIR22HG | 0.000 | 0.56 | IGHV3-30 | MIR22HG | 0.000 | 0.50 |
| CSF1R | MIR100HG | 0.000 | 0.56 | IGLV1-36 | MIR22HG | 0.000 | 0.50 |
| CMKLR1 | MIR100HG | 0.000 | 0.56 | IGLV2-8 | CDKN2B-AS1 | 0.000 | 0.50 |
| NPR1 | MIR100HG | 0.000 | 0.56 | IGHA1 | FENDRR | 0.000 | 0.50 |
| LTB4R | SNHG20 | 0.000 | 0.56 | PIK3CG | CARMN | 0.000 | 0.50 |
| SEMA3G | MIR100HG | 0.000 | 0.56 | PTPRC | MIR22HG | 0.000 | 0.50 |
| PI15 | AC104083.1 | 0.000 | 0.56 | IGLV5-45 | CDKN2B-AS1 | 0.000 | 0.50 |
| PRKCB | MIR22HG | 0.000 | 0.56 | PIK3CD | MAGI2-AS3 | 0.000 | 0.50 |
| FGF7 | MBNL1-AS1 | 0.000 | 0.56 | AGT | SNHG11 | 0.000 | 0.50 |
| GREM2 | MAGI2-AS3 | 0.000 | 0.56 | PLAU | CYTOR | 0.000 | 0.50 |
| PRKCB | MIR100HG | 0.000 | 0.56 | LCN12 | RUSC1-AS1 | 0.000 | 0.50 |
| SEMA3G | AC104083.1 | 0.000 | 0.56 | CSF2RB | AC104083.1 | 0.000 | 0.50 |
| CSF1 | CARMN | 0.000 | 0.56 | PRKCB | FENDRR | 0.000 | 0.50 |
| PLXNA1 | AC125807.2 | 0.000 | 0.56 | IGHV3-72 | FENDRR | 0.000 | 0.50 |
| TAFA5 | MIR100HG | 0.000 | 0.56 | BIRC5 | SNHG25 | 0.000 | 0.50 |
| EDNRB | MAGI2-AS3 | 0.000 | 0.56 | FAM3D | AC254629.1 | 0.000 | 0.50 |
| ZC3HAV1L | SNHG25 | 0.000 | 0.55 | CD22 | MAGI2-AS3 | 0.000 | 0.50 |
| PTGER3 | FENDRR | 0.000 | 0.55 | OLR1 | AC104083.1 | 0.000 | 0.50 |
| CSF1R | CARMN | 0.000 | 0.55 | TG | AC124067.4 | 0.000 | 0.50 |
| IGHV3-74 | MIR22HG | 0.000 | 0.55 | CCL28 | CDKN2B-AS1 | 0.000 | 0.50 |
| PRKCB | CARMN | 0.000 | 0.55 | PLXNA3 | MHENCR | 0.000 | 0.50 |
| FGFR4 | SNHG11 | 0.000 | 0.55 | NPR3 | MBNL1-AS1 | 0.000 | 0.50 |
| PIK3R5 | MIR22HG | 0.000 | 0.55 | VEGFA | AL355987.4 | 0.000 | 0.50 |
| MASP1 | MAGI2-AS3 | 0.000 | 0.55 | IGKV4-1 | CDKN2B-AS1 | 0.000 | 0.50 |
| IGLV3-19 | MIR22HG | 0.000 | 0.55 | SEMA6D | CDKN2B-AS1 | 0.000 | 0.50 |
| LTB4R | TMEM147-AS1 | 0.000 | 0.55 | NGFR | MAGI2-AS3 | 0.000 | 0.50 |
| A2M | MBNL1-AS1 | 0.000 | 0.55 | GREM2 | MBNL1-AS1 | 0.000 | 0.50 |
| PLXNA3 | AL390719.2 | 0.000 | 0.55 | ACVRL1 | AC254629.1 | 0.000 | 0.50 |
| PIK3R5 | CARMN | 0.000 | 0.55 | NPR1 | AC104083.1 | 0.000 | 0.50 |
| SCG2 | MBNL1-AS1 | 0.000 | 0.55 | ACVRL1 | MIR22HG | 0.000 | 0.50 |
| CSF2RB | MIR22HG | 0.000 | 0.55 | CCR7 | MIR22HG | 0.000 | 0.50 |
| LTB4R | LINC02604 | 0.000 | 0.55 | CCL19 | MAGI2-AS3 | 0.000 | 0.50 |
| VIP | CARMN | 0.000 | 0.55 | LCN12 | GAS6-AS1 | 0.000 | 0.50 |
| VIP | MAGI2-AS3 | 0.000 | 0.55 |  |  |  |  |

**Supplementary table 2 Differential infiltration analysis for tumor immune between normal and patients.**

| **Type** | **Cell** | **Mean** | **STD** | **Mean** | **STD** |
| --- | --- | --- | --- | --- | --- |
| CIBERSORT | Mast cell activated | 1.00 | 0.50 | 0.07 | 0.23 |
|  | B cell plasma | 1.00 | 0.59 | 0.38 | 0.44 |
|  | Macrophage M0 | 1.00 | 4.57 | 43.92 | 39.66 |
|  | Monocyte | 1.00 | 1.09 | 0.35 | 0.50 |
|  | Mast cell resting | 1.00 | 3.29 | 9.86 | 8.19 |
|  | T cell follicular helper | 1.00 | 0.92 | 2.25 | 1.47 |
|  | Macrophage M2 | 1.00 | 0.26 | 0.74 | 0.31 |
|  | Macrophage M1 | 1.00 | 1.85 | 1.97 | 1.43 |
|  | T cell CD4+ memory resting | 1.00 | 0.28 | 0.73 | 0.42 |
|  | NK cell resting | 1.00 | 2.12 | 4.52 | 5.95 |
|  | T cell CD4+ memory activated | 1.00 | 3.51 | 32.69 | 61.94 |
|  | T cell regulatory (Tregs) | 1.00 | 0.75 | 1.89 | 1.73 |
|  | B cell memory | 1.00 | 1.70 | 0.41 | 1.19 |
|  | B cell naive | 1.00 | 1.52 | 0.59 | 0.95 |
|  | Neutrophil | 1.00 | 3.75 | 4.42 | 8.76 |
| CIBERSORT-ABS | Mast cell activated | 1.00 | 0.48 | 0.07 | 0.25 |
|  | B cell plasma | 1.00 | 0.55 | 0.23 | 0.29 |
|  | T cell CD4+ memory resting | 1.00 | 0.39 | 0.45 | 0.36 |
|  | Monocyte | 1.00 | 1.11 | 0.23 | 0.46 |
|  | Macrophage M2 | 1.00 | 0.36 | 0.50 | 0.39 |
|  | Macrophage M0 | 1.00 | 4.84 | 21.34 | 23.91 |
|  | Mast cell resting | 1.00 | 3.35 | 5.27 | 5.60 |
|  | B cell naive | 1.00 | 1.74 | 0.34 | 0.79 |
|  | NK cell activated | 1.00 | 0.75 | 0.52 | 0.71 |
|  | T cell CD8+ | 1.00 | 0.71 | 0.59 | 0.66 |
|  | B cell memory | 1.00 | 1.69 | 0.31 | 1.06 |
|  | T cell CD4+ memory activated | 1.00 | 3.48 | 18.90 | 40.23 |
|  | Eosinophil | 1.00 | 2.76 | 0.29 | 1.43 |
|  | NK cell resting | 1.00 | 1.89 | 2.70 | 4.06 |
|  | Myeloid dendritic cell resting | 1.00 | 1.65 | 0.50 | 1.41 |
| EPIC | B cell | 1.00 | 0.89 | 0.21 | 0.37 |
|  | T cell CD4+ | 1.00 | 0.22 | 0.64 | 0.28 |
|  | Endothelial cell | 1.00 | 0.39 | 0.52 | 0.38 |
|  | T cell CD8+ | 1.00 | 0.42 | 0.61 | 0.42 |
|  | Macrophage | 1.00 | 0.47 | 0.57 | 0.53 |
|  | Cancer associated fibroblast | 1.00 | 0.54 | 3.43 | 4.74 |
| MCPCOUNTER | Neutrophil | 1.00 | 0.51 | 0.13 | 0.07 |
|  | Myeloid dendritic cell | 1.00 | 0.43 | 0.31 | 0.32 |
|  | B cell | 1.00 | 1.00 | 0.21 | 0.44 |
|  | NK cell | 1.00 | 0.40 | 0.27 | 0.48 |
|  | Endothelial cell | 1.00 | 0.29 | 0.66 | 0.38 |
|  | T cell | 1.00 | 0.38 | 0.72 | 0.42 |
|  | Monocyte | 1.00 | 0.40 | 0.68 | 0.55 |
|  | Macrophage/Monocyte | 1.00 | 0.40 | 0.68 | 0.55 |
|  | Cancer associated fibroblast | 1.00 | 1.66 | 0.56 | 0.68 |
| QUANTISEQ | uncharacterized cell | 1.00 | 0.16 | 1.42 | 0.10 |
|  | Neutrophil | 1.00 | 0.33 | 0.39 | 0.13 |
|  | T cell CD4+ (non-regulatory) | 1.00 | 0.40 | 0.25 | 0.25 |
|  | B cell | 1.00 | 0.98 | 0.26 | 0.42 |
|  | Macrophage M1 | 1.00 | 0.29 | 0.58 | 0.32 |
|  | T cell regulatory (Tregs) | 1.00 | 0.36 | 0.63 | 0.46 |
|  | Macrophage M2 | 1.00 | 1.56 | 0.55 | 0.34 |
|  | NK cell | 1.00 | 0.43 | 0.76 | 0.43 |
|  | Myeloid dendritic cell | 1.00 | 5.73 | 0.20 | 1.62 |
| TIMER | T cell CD8+ | 1.00 | 0.29 | 0.55 | 0.29 |
|  | B cell | 1.00 | 0.78 | 0.47 | 0.43 |
|  | Macrophage | 1.00 | 0.65 | 0.40 | 0.55 |
|  | Myeloid dendritic cell | 1.00 | 0.15 | 0.71 | 0.29 |
|  | T cell CD4+ | 1.00 | 0.34 | 0.69 | 0.36 |
|  | Neutrophil | 1.00 | 0.23 | 0.83 | 0.46 |
| XCELL | Class-switched memory B cell | 1.00 | 0.50 | 0.24 | 0.23 |
|  | B cell memory | 1.00 | 0.82 | 0.08 | 0.27 |
|  | microenvironment score | 1.00 | 0.38 | 0.30 | 0.35 |
|  | B cell | 1.00 | 0.96 | 0.16 | 0.36 |
|  | Plasmacytoid dendritic cell | 1.00 | 0.53 | 0.27 | 0.39 |
|  | B cell plasma | 1.00 | 0.58 | 0.39 | 0.31 |
|  | immune score | 1.00 | 0.58 | 0.32 | 0.37 |
|  | T cell NK | 1.00 | 0.38 | 0.46 | 0.31 |
|  | Common myeloid progenitor | 1.00 | 1.71 | 0.04 | 0.41 |
|  | Macrophage M1 | 1.00 | 0.33 | 0.38 | 0.45 |
|  | Eosinophil | 1.00 | 0.92 | 0.23 | 0.51 |
|  | Cancer associated fibroblast | 1.00 | 1.56 | 0.12 | 0.48 |
|  | Myeloid dendritic cell | 1.00 | 0.53 | 0.37 | 0.46 |
|  | Myeloid dendritic cell activated | 1.00 | 0.31 | 0.50 | 0.38 |
|  | T cell CD4+ Th1 | 1.00 | 1.25 | 12.82 | 9.35 |
|  | Mast cell | 1.00 | 0.65 | 0.48 | 0.37 |
|  | stroma score | 1.00 | 1.21 | 0.24 | 0.56 |
|  | Common lymphoid progenitor | 1.00 | 0.34 | 1.59 | 0.55 |
|  | Hematopoietic stem cell | 1.00 | 0.52 | 0.54 | 0.43 |
|  | Granulocyte-monocyte progenitor | 1.00 | 0.87 | 0.32 | 0.67 |
|  | T cell CD4+ Th2 | 1.00 | 1.46 | 3.46 | 2.83 |
|  | B cell naive | 1.00 | 2.19 | 0.14 | 0.79 |
|  | Neutrophil | 1.00 | 0.58 | 0.38 | 0.84 |
|  | T cell CD8+ central memory | 1.00 | 0.90 | 0.45 | 0.76 |
|  | Endothelial cell | 1.00 | 0.80 | 0.53 | 0.88 |
|  | Monocyte | 1.00 | 1.12 | 0.45 | 1.03 |
|  | T cell CD8+ naive | 1.00 | 0.62 | 0.77 | 0.47 |
|  | T cell gamma delta | 1.00 | 5.72 | 31.69 | 73.60 |
|  | Macrophage | 1.00 | 0.45 | 0.65 | 0.85 |
|  | T cell CD4+ central memory | 1.00 | 4.29 | 10.17 | 23.48 |
|  | T cell regulatory (Tregs) | 1.00 | 6.40 | 24.92 | 66.50 |
|  | T cell CD8+ | 1.00 | 1.33 | 0.64 | 1.01 |
|  | T cell CD4+ effector memory | 1.00 | 1.27 | 0.62 | 1.09 |

**Supplementary table 3 Functional analysis for DEGs for COAD in differential risk group.**

| Term | | Count | PValue | Genes |
| --- | --- | --- | --- | --- |
| BP | platelet degranulation | 4 | 0.0019 | EGF, IGF2, PPBP, F5 |
|  | maintenance of lens transparency | 2 | 0.0119 | FOXC1, MUC5AC |
| CC | extracellular space | 12 | 0.0002 | WNT10A, GNLY, EGF, CD109, IGF2, TFF2, PPBP, OLFM4, AMH, RAMP1, MUC5AC, F5 |
|  | platelet alpha granule lumen | 4 | 0.0003 | EGF, IGF2, PPBP, F5 |
|  | extracellular region | 11 | 0.0030 | WNT10A, LCN15, EGF, NXPH4, IGF2, PPBP, AMH, MTRNR2L1, MUC5AC, F5, MUC6 |
|  | extracellular exosome | 12 | 0.0496 | RAB3B, SCEL, STEAP4, EGF, REG1B, REG1A, IGF2, TFF2, AQP5, OLFM4, MUC5AC, KRT6A |
| MF | growth factor activity | 5 | 0.0005 | EGF, REG1A, IGF2, PPBP, AMH |

**
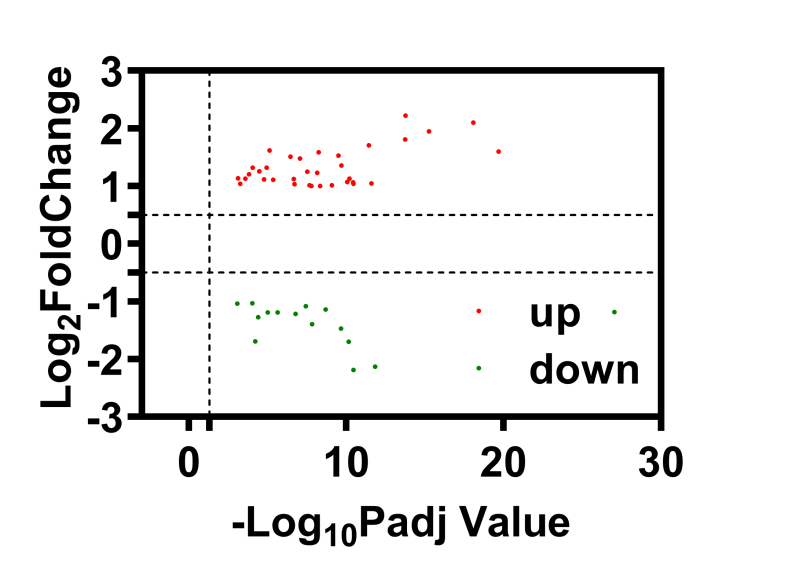
**

**Supplementary Figure 1 Volcano plot of DEGs for COAD in differential risk group.** Red, the upregulated DEGs in patients with COAD with high risk group. Green, the downregulated DEGs in patients with COAD with high risk group.

**
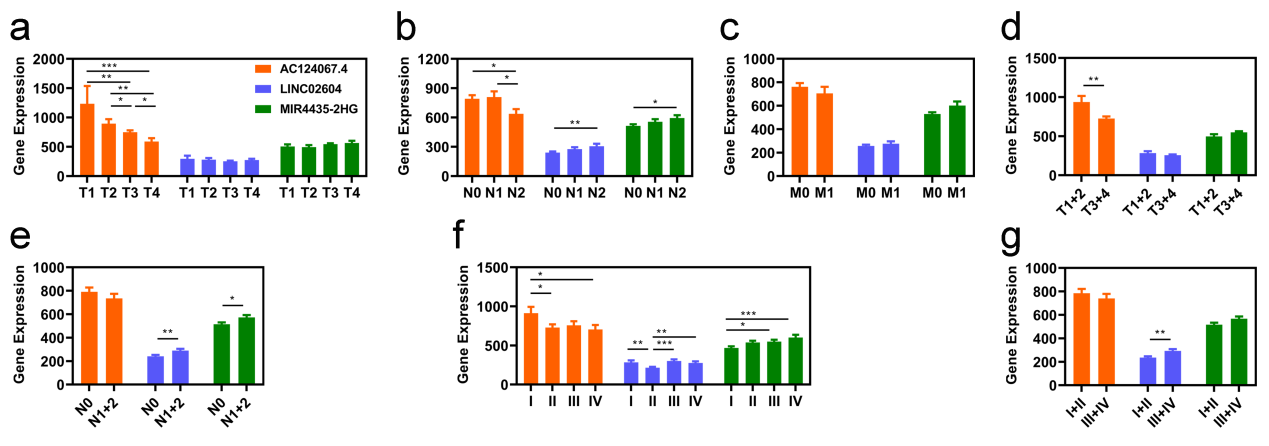
**

**Supplementary Figure 2 Expression of IR-DELs in difference pathologic TNM.**

Histograms showing that pathologic T (a, d), pathologic N (b, e), pathologic M (c), pathologic S (f, g). * means P <0.05, ** means P <0.01, *** means P <0.001
